# Antibiotic-Induced Alterations of the Gut Microbiota Alter Secondary Bile Acid Production and Allow for *Clostridium difficile* Spore Germination and Outgrowth in the Large Intestine

**DOI:** 10.1128/mSphere.00045-15

**Published:** 2016-01-06

**Authors:** Casey M. Theriot, Alison A. Bowman, Vincent B. Young

**Affiliations:** a Department of Population Health and Pathobiology, College of Veterinary Medicine, North Carolina State University, Raleigh, North Carolina, USA; b Department of Internal Medicine, Division of Infectious Disease, The University of Michigan Medical School, Ann Arbor, Michigan, USA; c Department of Microbiology and Immunology, The University of Michigan Medical School, Ann Arbor, Michigan, USA; University of Iowa

**Keywords:** *Clostridium difficile*, bile acids, antibiotics, microbiota, colonization resistance

## Abstract

It is hypothesized that the depletion of microbial members responsible for converting primary bile acids into secondary bile acids reduces resistance to *Clostridium difficile* colonization. To date, inhibition of *C. difficile* growth by secondary bile acids has only been shown *in vitro*. Using targeted bile acid metabolomics, we sought to define the physiologically relevant concentrations of primary and secondary bile acids present in the murine small and large intestinal tracts and how these impact *C. difficile* dynamics. We treated mice with a variety of antibiotics to create distinct microbial and metabolic (bile acid) environments and directly tested their ability to support or inhibit *C. difficile* spore germination and outgrowth *ex vivo.* Susceptibility to *C. difficile* in the large intestine was observed only after specific broad-spectrum antibiotic treatment (cefoperazone, clindamycin, and vancomycin) and was accompanied by a significant loss of secondary bile acids (deoxycholate, lithocholate, ursodeoxycholate, hyodeoxycholate, and ω-muricholate). These changes were correlated to the loss of specific microbiota community members, the *Lachnospiraceae* and *Ruminococcaceae* families. Additionally, physiological concentrations of secondary bile acids present during *C. difficile* resistance were able to inhibit spore germination and outgrowth *in vitro*. Interestingly, we observed that *C. difficile* spore germination and outgrowth were supported constantly in murine small intestinal content regardless of antibiotic perturbation, suggesting that targeting growth of *C. difficile* will prove most important for future therapeutics and that antibiotic-related changes are organ specific. Understanding how the gut microbiota regulates bile acids throughout the intestine will aid the development of future therapies for *C. difficile* infection and other metabolically relevant disorders such as obesity and diabetes.

**IMPORTANCE** Antibiotics alter the gastrointestinal microbiota, allowing for *Clostridium difficile* infection, which is a significant public health problem. Changes in the structure of the gut microbiota alter the metabolome, specifically the production of secondary bile acids. Specific bile acids are able to initiate *C. difficile* spore germination and also inhibit *C. difficile* growth *in vitro*, although no study to date has defined physiologically relevant bile acids in the gastrointestinal tract. In this study, we define the bile acids *C. difficile* spores encounter in the small and large intestines before and after various antibiotic treatments. Antibiotics that alter the gut microbiota and deplete secondary bile acid production allow *C. difficile* colonization, representing a mechanism of colonization resistance. Multiple secondary bile acids in the large intestine were able to inhibit *C. difficile* spore germination and growth at physiological concentrations and represent new targets to combat *C. difficile* in the large intestine.

## Abstract (precis)

Antibiotics alter the gastrointestinal microbiota, allowing for *Clostridium difficile* infection, which is a significant public health problem. Changes in the structure of the gut microbiota alter the metabolome, specifically the production of secondary bile acids. Specific bile acids are able to initiate *C. difficile* spore germination and also inhibit *C. difficile* growth *in vitro*, although no study to date has defined physiologically relevant bile acids in the gastrointestinal tract. In this study, we define the bile acids *C. difficile* spores encounter in the small and large intestines before and after various antibiotic treatments. Antibiotics that alter the gut microbiota and deplete secondary bile acid production allow *C. difficile* colonization, representing a mechanism of colonization resistance. Multiple secondary bile acids in the large intestine were able to inhibit *C. difficile* spore germination and growth at physiological concentrations and represent new targets to combat *C. difficile* in the large intestine.

## INTRODUCTION

*Clostridium difficile* infection (CDI) is a significant public health problem, associated with increasing morbidity, mortality, and health care-related costs in the United States and around the globe (1). Current treatment for patients with CDI includes the antibiotics vancomycin and metronidazole; however, even after successful treatment, this therapy is associated with more than 20% of cases relapsing (2–5). Even though antibiotics are the first line of treatment, they are also key risk factors in the pathogenesis of CDI (6, 7). Antibiotics alter the resident gut microbiota, decreasing resistance against *C. difficile* colonization (8–10). However, the exact mechanism for colonization resistance is still unknown.

By altering the gut microbiota, antibiotics ultimately change the gut metabolome (11, 12), specifically the composition and concentration of bile acids (11, 13–15). Bile acids are synthesized by hepatic enzymes from cholesterol and are important for lipoprotein, glucose, drug, and energy metabolism (16, 17). Mice synthesize two primary bile acids, cholate (CA) and β-muricholate (βMCA), whereas humans synthesize CA and chendeoxycholate (CDCA) (18). Bile acids are further conjugated with taurine and glycine (19). Once made in the liver, 95% of primary bile acids, both unconjugated and conjugated, are absorbed in the terminal ileum and through the hepatic system (16, 18). Primary bile acids that make it to the large intestine are biotransformed by members of the gut microbiota via two enzymatic reactions, deconjugation and dehydroxylation, into secondary bile acids, including ω-muricholate (ωMCA), hyodeoxycholate (HDCA), ursodeoxycholate, (UDCA), lithocholate (LCA), and deoxycholate (DCA) (16, 20, 21).

*C. difficile* is a spore-forming organism that requires specific bile acids for maximal germination into a metabolically active vegetative cell, where it can grow to high cell density and produce toxins (22–24). However, many microbially derived secondary bile acids inhibit *C. difficile* growth (22, 25, 26). Specific bile acids are able to enhance or inhibit *C. difficile* spore germination and vegetative cell outgrowth *in vitro*, and this is thought to be important for colonization resistance against *C. difficile*. Wilson et al. first suggested this concept in 1983, and more recent data support this hypothesis (25, 27–30). Multiple studies showing that restoration of secondary bile acids by members of the gut microbiota help restore colonization resistance against *C. difficile* in humans and in mice have been published recently (12, 28, 29). However, most studies defining the dynamics between *C. difficile* and bile acids have been done *in vitro*, and it is not clear how physiologically relevant this is *in vivo* (22). Based on our previous work and work by others, we hypothesize that antibiotics associated with decreased colonization resistance against *C. difficile* in the gut not only alter the gut microbiota but also decrease secondary bile acid pools, allowing for *C. difficile* spore germination and outgrowth. To test this hypothesis, we used a variety of antibiotics to create distinct microbial and metabolic (bile acid) environments in the murine gut and directly tested their ability to support or inhibit *C. difficile* spore germination and outgrowth *ex vivo.*

Here we show that susceptibility to *C. difficile* spore germination and outgrowth occurs in murine small intestinal content (ileum) regardless of antibiotic perturbation. Susceptibility to *C. difficile* spore germination and outgrowth in the large intestine (cecum) was present only after specific broad-spectrum antibiotic treatment (cefoperazone, clindamycin, and vancomycin) and was accompanied by a loss of secondary bile acids and significant changes to the gut microbiota. *In vivo* concentrations of secondary bile acids present during *C. difficile* resistance were able to inhibit spore germination and outgrowth *in vitro*. This study illustrates how antibiotics associated with increased risk of CDI are able to alter the gut microbiota, which more importantly results in a loss of secondary bile acid production, allowing for *C. difficile* colonization. Understanding how the gut microbiota regulates bile acids in both the small and large intestines is vital for designing future therapies to restore colonization resistance against *C. difficile* and for other metabolic disorders, including obesity and diabetes.

## RESULTS

### Small intestinal content supports *C. difficile* spore germination and outgrowth before and after antibiotic treatment.

**FIG 1:**
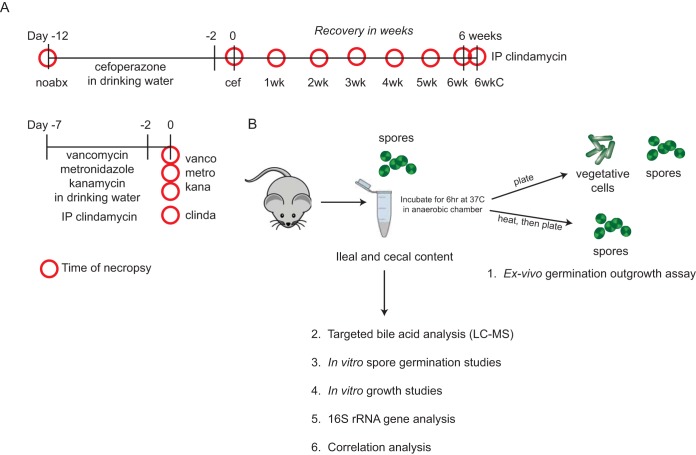
Antibiotic treatment scheme and experimental design. (A) C57BL/6 mice were treated with various antibiotics that would result in different microbial and metabolic environments. Red circles represent the time of necropsy for each group (*n =* 7 to 10 mice per group). (B) At the time of necropsy, ileal and cecal contents were collected. *Ex vivo* germination and outgrowth of *C. difficile* spores were measured in paired ileal and cecal contents from all treatment groups as well as by targeted bile acid analysis. *In vitro* spore germination and growth studies were done using relevant *in vivo* ileal and cecal bile acid concentrations. Microbiome analysis was also done to understand the relationship between gut bacteria and bile acids using correlation analysis. Abbreviations: noabx, no antibiotic; cef, cefoperazone; 1wk to 6wk, number of weeks off cefoperazone; 6wkC, 6 weeks off cefoperazone plus an intraperitoneal administration (IP) of clindamycin; vanco, vancomycin; metro, metronidazole; kana, kanamycin; clinda, clindamycin.

**FIG 2:**
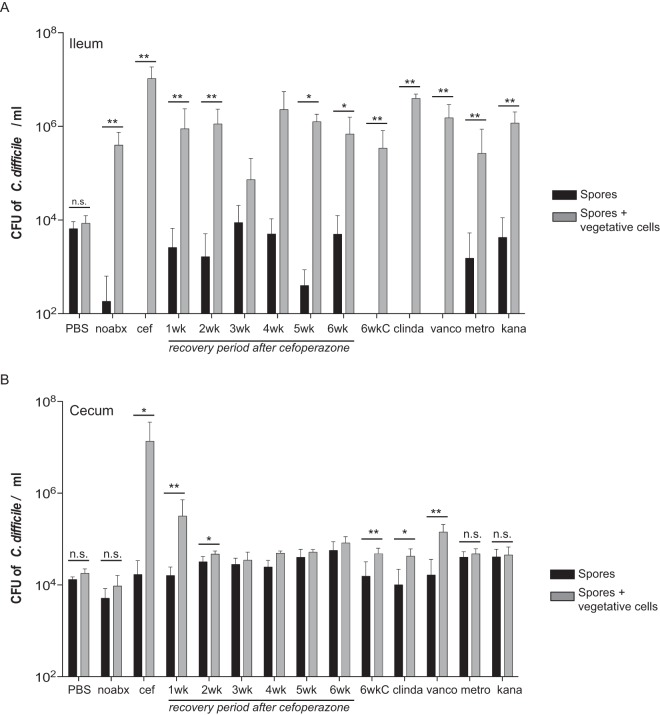
*C. difficile ex vivo* spore germination and outgrowth assays in murine ileal and cecal contents. *Ex vivo* germination and outgrowth of *C. difficile* spores were done in ileal (A) and cecal (B) contents collected from mice treated with various antibiotics. *C. difficile* VPI 10463 spores inoculated into the ileal contents of mice treated with or without antibiotics (noabx) allowed for spore germination and outgrowth after a 6-h period, whereas spores in non-antibiotic-treated cecal content did not. Only specific antibiotic treatments (cefoperazone [cef], 1 or 2 weeks off cefoperazone, 6 weeks off cefoperazone plus an intraperitoneal administration of clindamycin [6wkC], clindamycin [clinda], and vancomycin [vanco]) in the cecum supported spore germination and outgrowth. Black bars represent spores only, and gray bars represent spores and vegetative cells. Significance between groups was determined by Mann-Whitney nonparametric *t* test. Error bars represent the mean ± standard error of the mean (SEM) (*, *P* < 0.05; **, *P* < 0.01). n.s. not significant.

Groups of C57BL/6 mice were treated with various antibiotics (cefoperazone, clindamycin, vancomycin, metronidazole, and kanamycin), and some groups were allowed to recover off of the antibiotic cefoperazone for up to 6 weeks in order to create different microbial and metabolic environments in the small intestine (ileum) and the large intestine (cecum) (Fig. 1). At the time of necropsy (depicted by red circles in Fig. 1), ileal and cecal contents were collected for *ex vivo C. difficile* spore germination and outgrowth assays. After a 6-h incubation, *C. difficile* VPI 10463 spores (approximately 10^4^ spores/ml) were able to germinate and initiate outgrowth in ileal content from non-antibiotic-treated mice and almost all antibiotic-treated mice (Fig. 2A). Spores added to phosphate-buffered saline (PBS [negative control]) did not germinate or outgrow due to the lack of germinant present. The only two metabolic environments from ileal content that were able to initiate germination and outgrowth of spores but failed to reach significance were from mice allowed 3 and 4 weeks of recovery after cefoperazone.

### Large intestinal content supports *C. difficile* spore germination and outgrowth only after antibiotic treatment.

Unlike ileal content, *C. difficile* spores were only able to germinate and outgrow in cecal content from mice treated with specific antibiotics (Fig. 2B). We previously demonstrated non-antibiotic-treated mouse cecum does not support spore germination or outgrowth *ex-vivo*, whereas cefoperazone-treated mouse cecum does (12, 31). The only metabolic environments that allowed for significant spore germination and outgrowth in the mouse cecum *ex vivo* were directly after cefoperazone, 1 and 2 weeks of recovery after cefoperazone, 6 weeks of recovery after cefoperazone plus clindamycin, clindamycin alone, and vancomycin.

### Resistance to *C. difficile* germination and outgrowth is associated with secondary bile acids.

**FIG 3:**
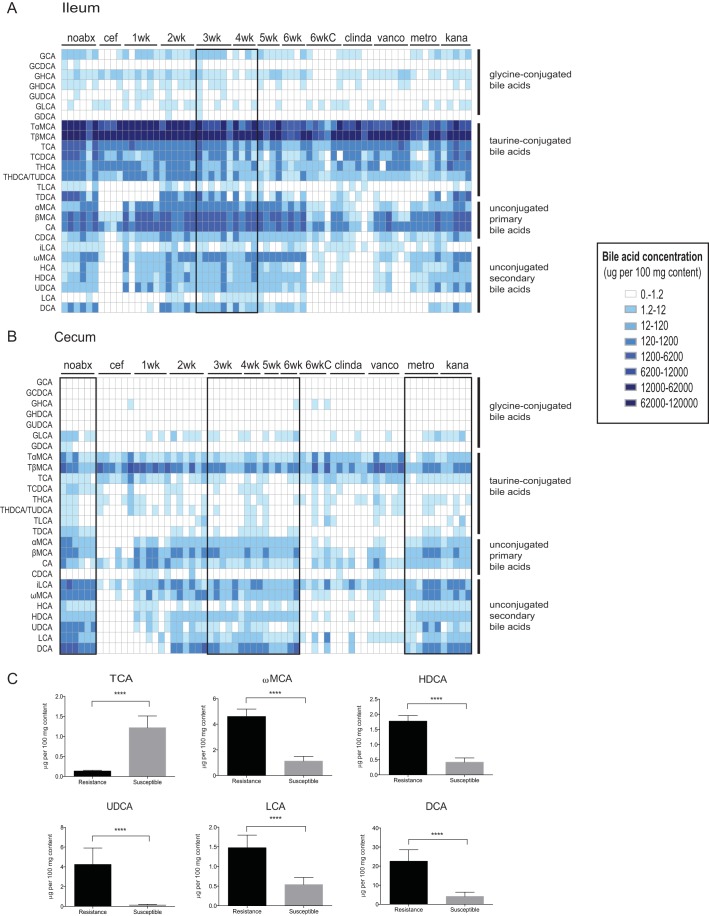
Targeted bile acid metabolomics of murine ileal and cecal contents. Bile acids were analyzed by LC-MS from paired ileal (A) and cecal (B) contents from *ex vivo* spore germination and outgrowth assays. A heat map shows the bile acid concentration present in micrograms per 100 mg of gut content, ranging from 1 to 120,000. The black boxes represent samples that did not reach significance in supporting spore germination and outgrowth of *C. difficile* spores in Fig. 2. (C) Bile acids present in cecal content that did not support spore germination and outgrowth in the black bars (resistance) or were able to support germination and outgrowth (susceptible) are compared. Significance between groups was determined by Mann-Whitney nonparametric *t* test. Error bars represent the mean ± SEM (****, *P* < 0.0001).

Since bile acids are required for *C. difficile* spore germination and are also able to inhibit outgrowth (22, 32), we defined the bile acids present in paired ileal and cecal samples from *ex vivo* germination and outgrowth assays. Prior to antibiotics, ileal content contains mostly primary bile acids, specifically taurine-conjugated and unconjugated primary bile acids, with a lesser concentration of unconjugated secondary bile acids (Fig. 3A). Primary bile acids taurocholate (TCA) and cholate (CA), both germinants of *C. difficile* spores, are always present at high concentrations in the ileum (average concentrations *in vivo*, TCA, 0.03%, and CA, 0.05%). This is in contrast to the cecum, where unconjugated secondary bile acids make up the majority of bile acids prior to antibiotics (Fig. 3B). Antibiotic treatments that supported *C. difficile* spore germination and outgrowth *ex vivo* in Fig. 2B (directly after cefoperazone, 1 and 2 weeks of recovery after cefoperazone, 6 weeks of recovery after cefoperazone plus clindamycin, clindamycin alone, and vancomycin) resulted in significantly more TCA and less secondary bile acids in the cecum (Fig. 3B and susceptible in panel C; see Fig. S1 and S2 in the supplemental material). Murine cecal contents that did not support *C. difficile* spore germination and outgrowth are highlighted in the black boxes in Fig. 3B and are associated with an increase in unconjugated secondary bile acids (resistant in Fig. 3C) (average concentrations *in vivo*, ωMCA, 0.004%, HDCA, 0.002%, UDCA, 0.004%, LCA, 0.001%, and DCA, 0.023%).

10.1128/mSphere.00045-15.1 Figure S1  Targeted bile acid metabolomics from murine ileal content. Bile acids were analyzed by LC-MS from paired ileal content from *ex vivo* spore germination and outgrowth assays. Bile acid concentrations are represented in micrograms per 100 mg of gut content from each treatment group. Antibiotic abbreviations: noabx, no antibiotic; cef, cefoperazone; 1 week to 6 weeks, weeks off of cefoperazone; 6wkC, 6 weeks off of cefoperazone plus an intraperitoneal administration of clindamycin; vanco, vancomycin; metro, metronidazole; kana, kanamycin; clinda, clindamycin. Download Figure S1, EPS file, 1.9 MB. Copyright © 2016 Theriot et al. 2016 Theriot et al. This content is distributed under the terms of the Creative Commons Attribution 4.0 International license.

10.1128/mSphere.00045-15.2 Figure S2  Targeted bile acid metabolomics from murine cecal content. Bile acids were analyzed by LC-MS from paired cecal contents from *ex vivo* spore germination and outgrowth assays. Bile acid concentrations are represented in micrograms per 100 mg of gut content from each treatment group. Download Figure S2, EPS file, 1.7 MB. Copyright © 2016 Theriot et al. 2016 Theriot et al. This content is distributed under the terms of the Creative Commons Attribution 4.0 International license.

We previously demonstrated using untargeted metabolomics that directly after cefoperazone treatment there was a significant decrease in unconjugated secondary bile acids in the murine cecum, and 6 weeks later, the levels returned to baseline or prior to antibiotic treatment (12). Here we show using a more sensitive targeted bile acid assay that secondary bile acids start to return to baseline levels within 2 weeks of recovery of cefoperazone, and by 6 weeks of recovery, they return to baseline levels (see Fig. S3 in the supplemental material).

10.1128/mSphere.00045-15.3 Figure S3  Antibiotics alter murine cecal bile acids. (A) Nonmetric multidimensional scaling (NMDS) illustrates dissimilarity indices via Horn distances between the bile acid profiles of mouse cecal samples labeled by antibiotic treatment. (B) Paired intestinal samples were used in *ex vivo* assays to determine if *C. difficile* spores were able to germinate and outgrow. Bile acid profiles that supported *C. difficile* germination and outgrowth are susceptible (red), and those that did not support germination and outgrowth are resistant (black). Download Figure S3, EPS file, 1.5 MB. Copyright © 2016 Theriot et al. 2016 Theriot et al. This content is distributed under the terms of the Creative Commons Attribution 4.0 International license.

### Secondary bile acids associated with resistance to *C. difficile* inhibit spore germination.

**FIG 4:**
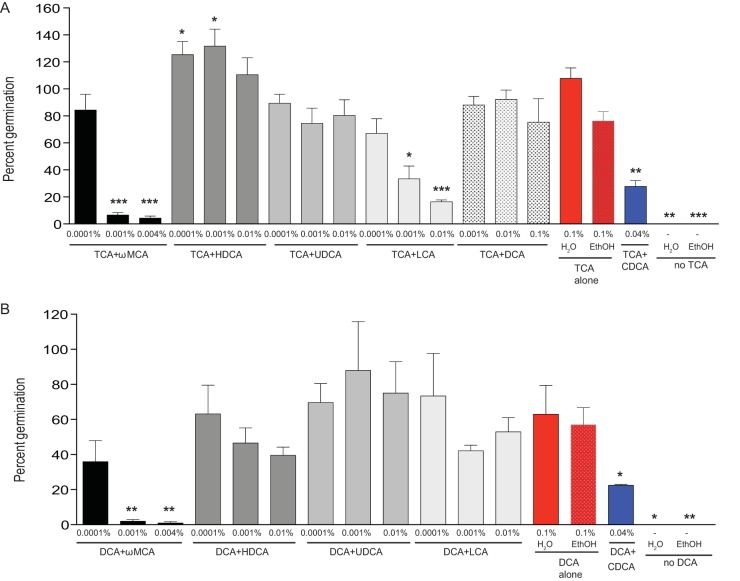
Secondary bile acids present during *C. difficile* resistance inhibit spore germination. *In vitro* spore germination inhibition assays were performed with *C. difficile* strain VPI 10463 to assess if the secondary bile acids ω-muricholate (ωMCA), hyodeoxycholate (HDCA), ursodeoxycholate (UDCA), lithocholate (LCA), and deoxycholate (DCA) were able to inhibit spore germination with known germinants TCA and DCA. Spores were incubated for 30 min in (A) BHI plus TCA (0.1%) or (B) BHI plus DCA (0.1%) supplemented with a range of secondary bile acids at relevant *in vivo* concentrations. Positive controls include BHI plus TCA (0.1%) or DCA (0.1%) alone with mock H_2_O and mock ethanol (EthOH) (red bars). A negative control was also used: BHI plus TCA supplemented with CDCA (0.04%) or BHI plus DCA supplemented with CDCA (0.04%), a known inhibitor of spore germination (blue bar). Negative controls include BHI alone without the addition of TCA or DCA. The data presented represent the mean ± standard deviation (SD) from triplicate experiments and were significant compared to the positive controls (A) TCA alone or (B) DCA alone (Student’s *t* test, *, *P* < 0.05; **, *P* < 0.01; ***, *P* < 0.001).

To define the relationship between relevant *in vivo* levels of secondary bile acids and colonization resistance to *C. difficile* in Fig. 2, we tested the ability of these bile acids to inhibit or enhance the first step of *C. difficile* colonization, spore germination. Previous studies have shown TCA is required for maximal spore germination, and chendeoxycholate (CDCA) is able to inhibit this interaction (26). Figure 4A shows maximal spore germination with TCA (0.1%) alone and significant inhibition of spore germination with the addition of CDCA (0.04%). Since most bile acids are soluble in ethanol (ωMCA, HDCA, UDCA, and LCA) a positive control of TCA made with ethanol was used for these comparisons. Secondary bile acids that significantly interfered with TCA-mediated spore germination in a concentration-dependent manner were ωMCA (0.001% and 0.004%) and LCA (0.001% and 0.01%), whereas HDCA (0.0001% and 0.001%) enhanced spore germination at lower concentrations.

Since the concentration of TCA (average concentration *in vivo* TCA, 0.0001%) is not high enough to support spore germination in the murine cecum during resistance to *C. difficile*, we turned our attention to the secondary bile acid DCA, which is highly abundant during resistance to *C. difficile* (average concentration *in vivo* DCA, 0.023%) and is a known germinant of *C. difficile* spores (12, 22). In Fig. 4B, spore germination with DCA (0.1%) alone does not reach the same maximal level of spore germination as TCA (0.1%) alone (Fig. 4A). The addition of CDCA (0.04%) significantly interfered with DCA-mediated spore germination as well as supplementation with ωMCA (0.001% and 0.004%). Secondary bile acids present in the cecum during resistance to *C. difficile* (ωMCA and LCA) were able to inhibit germination of *C. difficile* spores.

### Secondary bile acids associated with resistance to *C. difficile* inhibit growth.

**FIG 5:**
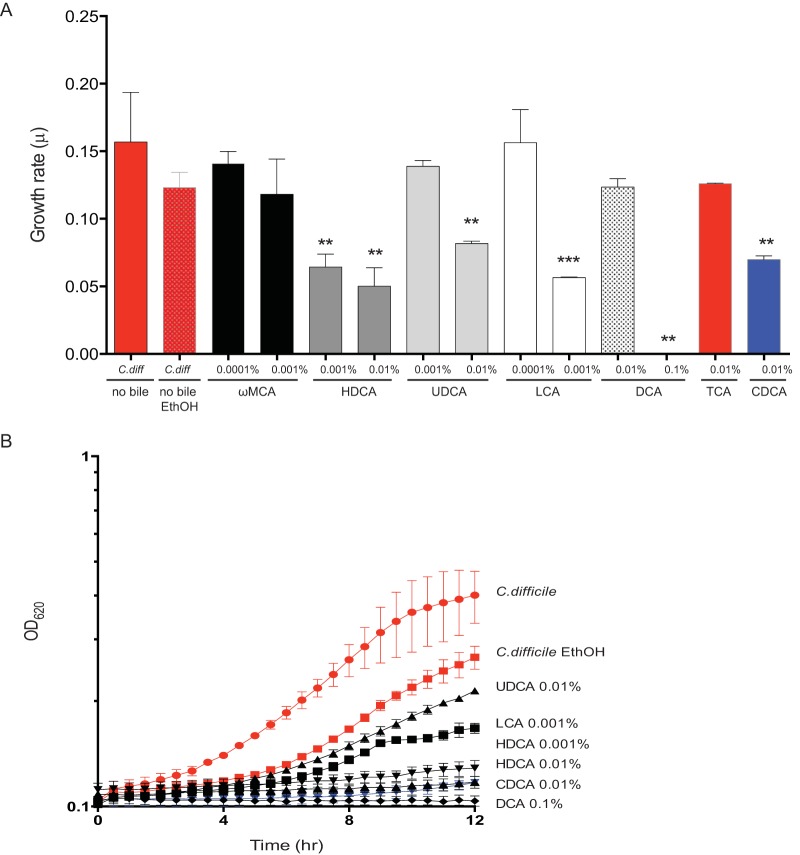
Secondary bile acids present during *C. difficile* resistance inhibit growth of *C. difficile*. (A) Growth rates (per hour) are shown. *C. difficile* was grown in BHI medium supplemented with various secondary bile acids with a range of *in vivo* concentrations (0.001% to 0.1%) present during resistance to *C. difficile*. The data presented represent the means ± SD from triplicate experiments and were significant compared to positive controls *C. difficile* and *C. difficile* with mock ethanol (EthOH) without bile acids (Students *t* test, **, *P* < 0.05; ***, *P* < 0.01). (B) Growth curve of representative secondary bile acids that significantly decreased the growth rate of *C. difficile* in panel A.

To further characterize the dynamics between the pathogen and secondary bile acids, we evaluated growth of *C. difficile* over a 24-h period *in vitro* in the presence of relevant *in vivo* concentrations of secondary bile acids. In media supplemented with secondary bile acids HDCA, UDCA, LCA, and DCA, *C. difficile* exhibited decreased growth rates compared to controls (*C. difficile* alone and *C. difficile* with ethanol added and no bile) without the addition of secondary bile acids. (Ethanol was added to a *C. difficile* culture to account for the ethanol in the bile acids.) *C. difficile* growth curves with secondary bile acids that significantly inhibited the growth rate from Fig. 5A are displayed in Fig. 5B (see Fig. S4 in the supplemental material). Secondary bile acids present during resistance to *C. difficile* were able to inhibit growth of *C. difficile*.

10.1128/mSphere.00045-15.4 Figure S4  *C. difficile* growth curves supplemented with secondary bile acids. (A) *In vitro* growth of *C. difficile* strain VPI 10463 was done in BHI medium over a 12-h period anaerobically. BHI medium was supplemented with various secondary bile acids (black line), with a range of *in vivo* concentrations (0.001% to 0.1%) present during resistance to *C. difficile*. The data presented represent the mean ± SD from triplicate experiments. Positive controls include *C. difficile* (red line) and *C. difficile* with mock ethanol (EthOH) (red line) without bile acids. Download Figure S4, EPS file, 1.3 MB. Copyright © 2016 Theriot et al. 2016 Theriot et al. This content is distributed under the terms of the Creative Commons Attribution 4.0 International license.

### Members of the *Lachnospiraceae* and *Ruminococcaceae* families in the large intestinal microbiota are positively correlated with secondary bile acids.

**FIG 6:**
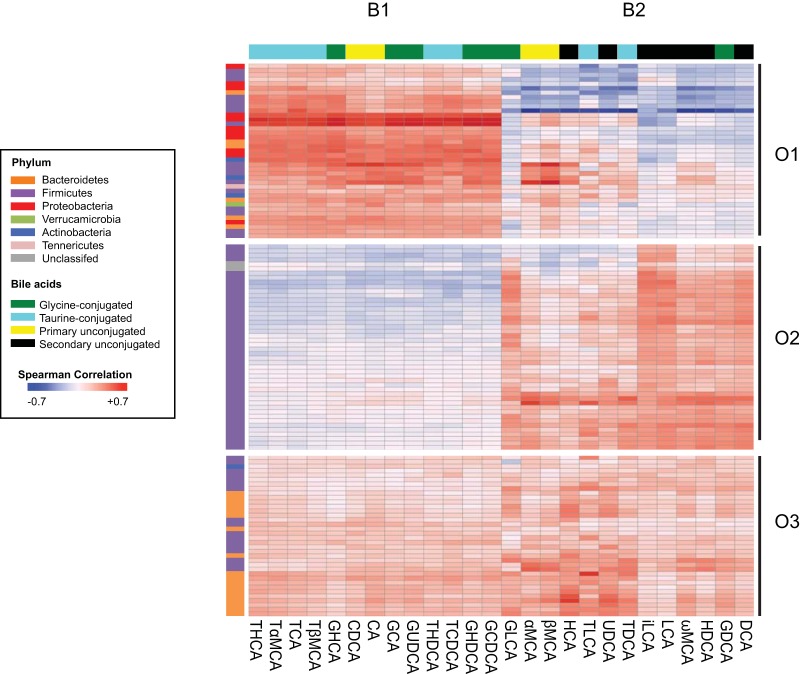
Correlation analysis of the gut microbiome and bile acids. Spearman’s correlation analysis was done with all 121 OTU (i.e., OTU that were greater than 1% of the total bacterial population) in the microbiome, color coded by phylum and grouped based on unsupervised clustering. All 26 bile acids detected were similarly clustered and are color coded based on structure. There were three distinct clusters of OTU (O1 to O3) and two distinct clusters of bile acids (B1 and B2). The heat map scale ranges from positively correlated, +0.7, to negatively correlated, −0.7.

Since many secondary bile acids shape resistance to *C. difficile*, and members of the gut microbiota are responsible for the biotransformation of primary bile acids to secondary bile acids, we defined the microbiome of each ileal and cecal sample (see Fig. S5 and S6 in the supplemental material). To examine the relationship between members of the gut microbiota and bile acids, we calculated the Spearman’s rank correlation coefficient for all operational taxonomic units (OTU) within the microbiome and bile acids using data from the mouse ileum and cecum (Fig. 6). To visualize these correlations, we performed unsupervised clustering of OTU and bile acids from all groups, which revealed three distinct OTU clusters (O1 to O3 in Table S1 in the supplemental material) and two bile acid clusters (B1 and B2). The organization of the correlation revealed distinctive relationships between OTU and bile acids in the different groups. OTU in the first OTU cluster (O1) were positively correlated with bile acids in the first bile acid cluster (B1) and negatively correlated with most of the bile acids in cluster B2, which contains all of the secondary bile acids (black boxes in Fig. 6). The OTU in cluster O1 include many members from the *Proteobacteria* and *Firmicutes* phyla, more specifically from the *Enterobacteriaceae* and *Lactobacillaceae* families (see Table S1). Many of these bacteria were present in samples that supported *C. difficile* germination and outgrowth. This is in contrast to the relationship between clusters O2 and B2, which has a positive correlation. O2 is made up of members from the *Firmicutes* phylum: specifically, these *Lachnospiraceae* and *Ruminococcaceae* family members are positively correlated with all secondary bile acids (see Table S1). Many of these bacteria were present in samples that provided resistance against *C. difficile* germination and outgrowth. Cluster O3 is made up of many members from the *Bacteroidetes* phylum, from the *Porphyromonadaceae* family (see Table S1) and is positively correlated with all bile acids from clusters B1 and B2.

10.1128/mSphere.00045-15.5 Figure S5  Structural changes to the murine ileal and cecal microbiome. A heat map of the top 121 operational taxonomic units (OTU) (i.e., OTU that were >1% of the total bacterial population) in the same orientation as Fig. 6. The bacterial phyla are listed by color, and the scale ranges from 0 to 100% relative OTU abundance. The black boxes represent samples that did not reach significance in supporting spore germination and outgrowth of *C. difficile* spores in Fig. 2. Download Figure S5, JPG file, 0.9 MB. Copyright © 2016 Theriot et al. 2016 Theriot et al. This content is distributed under the terms of the Creative Commons Attribution 4.0 International license.

10.1128/mSphere.00045-15.6 Table S1  Structural changes to the murine ileal and cecal microbiome. OTU are listed in order from Fig. 6 (and see Fig. S5 in the supplemental material) as displayed by taxonomic classification. Download Table S1, XLS file, 0.05 MB. Copyright © 2016 Theriot et al. 2016 Theriot et al. This content is distributed under the terms of the Creative Commons Attribution 4.0 International license.

## DISCUSSION

Using a targeted metabolomics approach, we defined the *in vivo* concentrations of bile acids before and after various antibiotic treatments in the murine small and large intestines. *C. difficile* spores were able to germinate and outgrow in most ileal content and cecal content that was depleted of secondary bile acids and had significant alterations to the microbiome. To further define the mechanism of colonization resistance against *C. difficile*, we conducted *in vitro* studies to show that *in vivo* concentrations of secondary bile acids were able to inhibit spore germination and growth in the large intestine. Previous *in vitro* studies looking at the interaction between bile acids and *C. difficile* spore germination and growth observed inhibition of germination with ωMCA, LCA, and UDCA and inhibition of growth with ωMCA, LCA, and DCA (12, 22, 25, 26, 28, 32, 33). However, they were not based on *in vivo* bile acid levels in the gastrointestinal (GI) tract. Newer, more sensitive mass spectrometric technology has allowed us to define the real-time physiological concentrations *in vivo* as opposed to untargeted metabolomic approaches, which only yield relative abundance (34). Defining the composition and concentration of bile acids that are able to inhibit or enhance *C. difficile* spore germination and outgrowth in the gut is critical for the development of targeted bacterial therapeutics to prevent *C. difficile*.

Interestingly, we observed that *C. difficile* spores were able to germinate in ileal content of the mouse before and after antibiotic treatment. Germination has been demonstrated in the small intestine in other rodent models (31, 35, 36). Although primary bile acids are absorbed in the small intestine, it is likely that sufficient levels are present to allow some level of spore germination. We detected consistently high levels of the germinants TCA and CA, even in the absence of antibiotic treatment, a finding that has been reported previously (14). It is hard to say what the concentration of primary bile acids is in the human small intestine because collection of such samples is difficult, although we know that human serum is rich in primary bile acids, which are mostly absorbed in the small intestine (37).

It is also challenging to say what the oxygen content is in the small intestine. Multiple studies measuring oxygen tension in the small intestine of rats, sheep, ducks, and mice show a spatial distribution, where the mucosa is more oxygen rich and the lumen is more anaerobic (38–40). More recent literature using sensitive oxygen-measuring imaging found the ileum in a mouse, which is relevant to our model, to be quite anaerobic and to resemble that of the colon (38). Similarly, the oxygen concentrations in the duck small intestinal lumen measured by microelectrodes were 25 mm Hg closest to the villi and <0.5 mm Hg in the center of the lumen (39). This further supports that the luminal content of the ileum is anaerobic. This study and our previous work also suggest that spores will always germinate to some degree in small intestinal content (31). Therefore, prevention of *C. difficile* from growing in the cecum and large intestine will be critical.

In the cecum, we observed that specific antibiotic treatments (cefoperazone, vancomycin, and clindamycin) altered the gut microbiome and decreased the secondary bile acid load, allowing *C. difficile* to germinate and outgrow. Some of these antibiotics are associated with susceptibility to CDI, with the highest risk associated with cephalosporin, clindamycin, penicillin, and fluoroquinolones (7, 41, 42). Interestingly, vancomycin is the preferred treatment for CDI but has been shown to alter the microbiome, bile acid metabolism, and host physiology in both mice and humans (13, 15, 43–45).

In our study, bacterial members from the *Firmicutes* phylum, specifically the *Lachnospiraceae* and *Ruminococcaceae* families, were positively correlated with secondary bile acids in the cecum and resistance to *C. difficile*. A small subset of spore-forming, anaerobic members of the class *Clostridia* are able to perform enzymatic reactions on conjugated bile acids, including deconjugation and 7α-dehydroxylation, which is a multistep biotransformation from CA to DCA and CDCA to LCA (16, 17, 20, 46). Well-characterized bacteria that have 7α-dehydroxylation activity are from the *Clostridium* species and include *Clostridium scindens*, *Clostridium hiranonis*, *Clostridium hylemonae*, and *Clostridium sordellii*, which belong to the *Blautia*, *Ruminococcaceae*, and *Lachnospiraceae* families (46–48). Most recently there is renewed interest in *C. scindens* because of its high 7α-dehydroxylation activity and its presence in patients’ resistant to *C. difficile* (25, 28). Future therapies to restore colonization resistance against *C. difficile* could potentially include targeted bacterial cocktails that are able to restore the level of secondary bile acids in the large intestine to inhibit *C. difficile* (28, 49). Much attention has focused on increasing DCA levels in the gut. However, increased levels of DCA are associated with an increased risk of colon cancer (50, 51). This study identifies new secondary bile acid targets (LCA, UDCA, HDCA, and ωMCA) at inhibitory concentrations, which could be produced by bacterial therapies to inhibit *C. difficile*.

Bacterial members from the *Proteobacteria* and *Firmicutes* phyla, more specifically from the *Enterobacteriaceae* and *Lactobacillaceae* families, were negatively correlated with secondary bile acids in this study. This is consistent with another study in which blooms of proinflammatory *Enterobacteriaceae* were found in cirrhotic patients and corresponded with a decrease in fecal bile acid levels (52). It is not known whether members from the *Proteobacteria* phylum are sensitive to the antimicrobial properties of secondary bile acids, but it has been suggested that they may limit their growth in the gut. Members of the *Lactobacillaceae* family include many *Lactobacillus* species, and they contain potent bile salt hydrolases, which are able to deconjugate glycine- or taurine-conjugated bile acids into unconjugated bile acids (53, 54). The lack of bacteria able to make secondary bile acids after antibiotics would cause a buildup of primary bile acids like TCA and CA, which are germinants of *C. difficile* spores.

More recently, human studies have shown recovery of fecal secondary bile acids, and members from the class *Clostridia*, including members from the *Lachnospiraceae* family, were associated with successful fecal transplantation in patients with recurrent CDI (29, 55). Even though this is promising, spore germination and colonization occurs upstream of the feces. The current standard of measuring the gut microbiota structure is via feces collection, and it is difficult to collect samples from the more relevant upper human GI tract for studying *C. difficile*. Although the bile acid profile of mice differs from that of humans, an animal model of CDI allows for the assessment of bile acid concentrations throughout the GI tract (31). More promising is the observation that more sensitive bile acid metabolomics, such as liquid chromatography-tandem mass spectrometry (LC-MS/MS) assays, are seeing similar bile acid species in both human and mouse sera (37), suggesting the usefulness of this mouse model.

Finally, there are potential limitations to the present study, including the use of *C. difficile* VPI 10463, which is still used in many studies today, although it is not a clinically relevant strain (8, 28, 31). This strain still provides us with a reproducible model of *C. difficile* infection in the mouse. Moving forward, these observations need to be validated with clinically relevant strains as they use a wide range of bile acids for germination (56, 57). The *ex vivo* approach we used is powerful, but it does not account for transit time in the GI tract, as it is a static sample. Even though bile acids are able to inhibit spore germination and the growth rate of *C. difficile* in the gut, bile acids do not represent the sole mechanism for colonization resistance against *C. difficile*. Other factors that could contribute to colonization resistance in the gut and in our *ex vivo* samples include competition for nutrients by other members of the gut microbiota.

Since alterations in the gut microbiome and bile acid metabolism are associated with many diseases, including diabetes, obesity, cancer, and metabolic syndrome (21, 50, 51, 58), further investigation is needed to understand the complex relationship between the gut microbiota, bile acids, and host physiology.

## MATERIALS AND METHODS

### Ethics statement.

The University Committee on the Use and Care of Animals (UCUCA) at the University of Michigan approved this study. The University of Michigan laboratory animal care policies follow the *Public Health Service Policy on Humane Care and Use of Laboratory Animals* (59). Animals were assessed twice daily for physical condition and behavior, and those assessed as moribund were humanely euthanized by CO_2_ asphyxiation. Trained animal technicians performed animal husbandry in an AAALAC-accredited facility.

### Animals and housing.

Five- to 8-week-old C57BL/6 wild-type (WT) mice (male or female) were obtained from a breeding colony at the University of Michigan that was originally established using animals purchased from Jackson Laboratories (Bar Harbor, ME). Mice were housed with autoclaved food, bedding, and water. Cage changes were performed in a laminar flow hood. Mice had a 12-h cycle of light and darkness.

### Antibiotic treatment of animals and sample collection.

Groups of C57BL/6 WT mice (male and female) ranging from 5 to 8 weeks in age were treated with a variety of antibiotics (Fig. 1). Groups of mice (*n =* 7 to 10 per group) were given cefoperazone (0.5 mg/ml) in sterile drinking water for 10 days followed by a recovery period of 2 days and 1, 2, 3, 4, 5, and 6 weeks with regular drinking water. Three additional groups of mice were treated with a 5-day course of vancomycin (0.5 mg/ml), metronidazole (0.5 mg/ml), or kanamycin (0.5 mg/ml) in their drinking water followed by a 2-day recovery period with regular drinking water. Mice treated with clindamycin were given a subcutaneous injection of 1 mg/ml clindamycin solution so that each mouse received 10 mg/kg of body weight followed by a 1-day recovery. An additional group of mice was given an injection of clindamycin after 6 weeks of recovery from a 10-day treatment with cefoperazone (0.5 mg/ml) followed by a 1-day recovery after the clindamycin injection. Non-antibiotic-treated controls for this experiment included mice given sterile drinking water during the duration of these experiments. After each treatment, mice were euthanized, and at the time of necropsy, ileal and cecal contents were collected, flash-frozen in liquid nitrogen, and stored at −80°C until further analysis.

### Bile acid analysis of mouse ileal and cecal contents. (i) Sample preparation.

A two-step extraction of gut content with ethanol and 1:1 chloroform-methanol was done with isotope-labeled internal standards spiked in. Extraction solvent was added to gut samples for homogenization with a probe sonicator. Homogenized samples were kept on ice for 10 min and centrifuged at 45°C at 13000 rpm for 10 min. Supernatants containing metabolites were transferred to another tube, and pellets were extracted with 1:1 chloroform-methanol. Supernatants from the two steps were combined and dried under vacuum at 45°C by using a vacuum centrifuge. Dried samples were reconstituted in 50:50 methanol-water for LC-MS analysis. A series of calibration standards were prepared and analyzed along with samples to quantify metabolites.

### (ii) LC-MS analysis.

An Agilent 1290 liquid chromatograph (Agilent, Santa Clara, CA) and waters Acquity BEH C_18_ column with 1.7-µm particle size (2.1 by 100 mm; Waters, Milford, MA) was used for chromatographic separation. Mobile phase A (MPA) was 0.1% formic acid in LC-MS-grade water. Mobile phase B (MPB) was 0.1% formic acid in acetonitrile. The mobile phase gradient consisted of 0 to 0.5 min of 5% MPB, 3 min of 25% MPB, 17 min of 40% MPB, and 19 to 21 min of 95% MPB and was run on an Agilent 6490 triple-quadrupole mass spectrometer (Agilent, Santa Clara, CA). Data were processed by MassHunter workstation software, version B.06. Table 1 lists the bile acids detected by name and abbreviation as well as their MRM transitions (i.e., parent *m*/*z*→daughter *m*/*z*).

**TABLE 1:** Bile acids identified by LC-MS analysis

Name	Abbreviation	MRM transition^a^
α-Muricholate	αMCA	407.3→407.3
β-Muricholate	βMCA	407.3→407.3
ω-Muricholate	ωMCA	407.3→407.3
Hyocholate (γ-muricholate)	HCA (γ-MCA)	407.3→407.3
Cholate	CA	407.3→407.3
Chenodeoxycholic acid	CDCA	391.3→391.3
Deoxycholic acid	DCA	391.3→391.3
Ursodeoxycholic acid	UDCA	391.3→391.3
Hyodeoxycholate	HDCA	391.3→391.3
Glycocholic acid	GCA	464.3→74.0
Glycochenodeoxycholate	GCDCA	448.3→74.0
Glycodeoxycholate	GDCA	448.3→74.0
Glycoursodeoxycholate	GUDCA	448.3→74.0
Glycohyodeoxycholate	GHDCA	448.3→74.0
Glycolithocholate	GLCA	432.3→74.0
Lithocholic acid	LCA	375.3→375.3
Taurocholic acid	TCA	514.3→80.0
Taurochenodeoxycholate	TCDCA	498.3→80.0
Taurodeoxycholate	TDCA	498.3→80.0
Tauroursodeoxycholate	TUDCA	498.3→80.0
Taurohyodeoxycholate/tauroursodeoxycholate	THDCA/TUDCA	498.3→80.0
Taurolithocholate	TLCA	482.3→80.0
Tauro-β-muricholic acid	T-βMCA	514.3→80.0
Taurohyocholate	THCA	514.3→80.0
Tauro-α-muricholic acid	TαMCA	514.3→80.0
Glycohyocholic acid	GHCA	464.3→74.0

^a^ Parent *m*/*z*→daughter *m*/*z*.

### *C. difficile* spore preparation.

*C. difficile* VPI 10463 spores were prepared as described in previous studies (12, 60).

### *Ex vivo* spore germination and outgrowth studies in mouse ileal and cecal contents.

*C. difficile* spores were subjected to heat treatment (65°C for 20 min) prior to use for these studies. Mouse ileal and cecal contents (non-antibiotic treated and antibiotic treated) were weighed and passed into an anaerobic chamber, diluted at a 1:1 ratio with PBS, and spun down for 5 s in a microcentrifuge. Approximately 10^3^ *C. difficile* spores (10^4^ spores/ml) were added to diluted ileal content, cecal content, and a PBS-only control at a final volume of 0.1 ml and then incubated at 37°C anaerobically for 6 h. After incubation, bacterial enumeration was done on taurocholate-cycloserine-cefoxitin-fructose agar (TCCFA) selective medium (12). The remaining ileal and cecal contents were heat treated at 65°C for 20 min to kill off any vegetative cells, and a subsequent bacterial enumeration was done. Samples plated prior to heating represent spores and vegetative cells (germination and outgrowth). Samples plated after heat treatment represent the spores remaining.

### *C. difficile in vitro* germination assays with secondary bile acids.

Spores were subjected to heat treatment (65°C for 20 min) prior to use for these studies. Sterile bile acid solutions dissolved in either water or ethanol were prepared and passed into an anaerobic chamber and added to brain heart infusion (BHI) plus 100 mg/liter l-cysteine broth with either 0.1% taurocholate (TCA) or 0.1% deoxycholate (DCA) as a positive control. *C. difficile* spores were added to broth and allowed to incubate for 30 min at 37°C anaerobically. Bacterial enumeration of the samples was performed on both BHI plates (vegetative cells) and BHI agar supplemented with 0.1% TCA (total CFU). Since 0.04% chendeoxycholate (CDCA) is a known inhibitor of TCA-mediated spore germination, this was used as a negative control in this assay (32). TCA at 0.1% and DCA at 0.1% made in water or ethanol (10%) were used as positive controls of spore germination. The percentage of germination of the positive controls TCA and DCA alone was calculated as [(CFU on BHI)/(CFU on BHI + TCA or DCA)] × 100. Inhibition of germination with TCA with the addition of secondary bile acids ωMCA, HDCA, UDCA, LCA, and DCA was calculated as [(CFU on BHI + TCA + secondary bile acids)/(CFU on BHI + TCA alone)] × 100. Inhibition of germination with DCA with the addition of secondary bile acids ωMCA, HDCA, UDCA, and LCA was calculated as [(CFU on BHI + DCA + secondary bile acids)/(CFU on BHI + DCA alone)] × 100. Germination under each condition was performed in triplicate. Controls were always used and compared to the proper bile acid backgrounds of water or ethanol.

### *C. difficile in vitro* growth curves with secondary bile acids.

*C. difficile* VPI 10463 was cultured overnight at 37°C in BHI plus 100 mg/liter·l-cysteine broth in an anaerobic growth chamber (Coy labs). After 14 h of growth, *C. difficile* was subcultured 1:10 into BHI plus 100 mg/liter·l-cysteine and allowed to grow for 3 h anaerobically at 37°C. The culture was then diluted in fresh BHI so that the starting optical density at 620 nm (OD_620_) was 0.01. The cell suspension was added to a 96-well plate at a final volume of 0.2 ml. Sterile bile acid solutions were added to the wells in triplicate. Cell density (OD_620_) was monitored every 30 min for 24 h, shaking the plate for 90 s before each reading, in a Tecan plate reader (reference no. 16039400) inside an anaerobic chamber. The specific growth rate was determined as μ = ln(*X*/*X_o_*)/*T*, where *X* is the OD_620_ value during the linear portion of growth and *T* is time in hours. Values given are representative of the mean μ values from two independent cultures done in triplicate. The growth curve data in Fig. S5 in the supplemental material start at an OD of 0.1 because BHI medium alone has a starting OD_620_ of 0.1.

The bile acids used in all *in vitro* assays include taurocholic acid sodium salt hydrate (TCA) (Sigma-Aldrich, CAS no. 345909-26-4), sodium deoxycholate (DCA) (Sigma-Aldrich, CAS no. 302-9504), chenodeoxycholic acid (CDCA) (Alfa Aesar, CAS no. 474-25-9), lithocholic acid (LCA) (Sigma-Aldrich, CAS no. 434-13-9), ursodeoxycholic acid (UDCA) (Sigma-Aldrich, CAS no. 128-13-2), ω-muricholic acid (ωMCA) (Steraloids, catalog ID no. C1888-000), hyocholic acid (HCA) (Steraloids, CAS no. 547-75-1), and hyodeoxycholic acid (HDCA) (Steraloids, CAS no. 83-49-8). TCA and DCA were soluble in water, and the rest of the bile acids were soluble in ethanol.

### Illumina MiSeq sequencing of bacterial communities.

Microbial DNA was extracted from mouse ileal and cecal tissue snips that also included luminal content using the PowerSoil-htp 96-well soil DNA isolation kit (Mo Bio Laboratories, Inc.). The V4 region of the 16S rRNA gene was amplified from each sample using a dual-indexing sequencing strategy (61). Each 20-µl PCR mixture contained 2 µl of 10× Accuprime PCR buffer II (Life Technologies), 0.15 µl of Accuprime high-fidelity *Taq* (catalog no. 12346094) high-fidelity DNA polymerase (Life Technologies), 2 µl of a 4.0 µM primer set, 1 µl DNA, and 11.85 µl sterile double-distilled water (ddH_2_O) (free of DNA, RNase, and DNase contamination). The template DNA concentration was 1 to 10 ng/µl for a high bacterial DNA/host DNA ratio. PCR was performed under the following conditions: 2 min at 95°C, followed by 30 cycles of 95°C for 20 s, 55°C for 15 s, and 72°C for 5 min, followed by 72°C for 10 min. For low-biomass samples (ileal content), a “touchdown PCR” protocol was performed. Each 20-µl PCR mixture contained 2 µl of 10× Accuprime PCR buffer II (Life Technologies), 0.15 µl of Accuprime high-fidelity *Taq* (catalog no. 12346094) high-fidelity DNA polymerase (Life Technologies), 2 µl of 4.0 µM primer set, 1 µl DNA, and 11.85 µl sterile ddH_2_O (free of DNA, RNase, and DNase contamination). The template DNA concentration was 1 to 10 ng/µl for a high bacterial DNA/host DNA ratio. PCR was performed under the following conditions: 2 min at 95°C, followed by 20 cycles of 95°C for 20 s, 60°C for 15 s, and 72°C for 5 min (with a 0.3°C increase of the 60°C annealing temperature each cycle), followed by 20 cycles of 95°C for 20 s, 55°C for 15 s, and 72°C for 5 min, followed by 72°C for 10 min. Libraries were normalized using a Life Technologies SequalPrep normalization plate kit (catalog no. A10510-01) following the manufacturer’s protocol. The concentration of the pooled samples was determined using the Kapa Biosystems library quantification kit for Illumina platforms (KapaBiosystems KK4854). The sizes of the amplicons in the library were determined using the Agilent Bioanalyzer high-sensitivity DNA analysis kit (catalog no. 5067-4626). The final library consisted of equal molar amounts from each of the plates, normalized to the pooled plate at the lowest concentration.

Sequencing was done on the Illumina MiSeq platform, using a MiSeq reagent kit V2 with 500 cycles (catalog no. MS-102-2003) according to the manufacturer’s instructions, with modifications (61). Libraries were prepared according to Illumina’s protocol for preparing libraries for sequencing on the MiSeq (part 15039740 Rev. D) for 2 or 4 nM libraries. The final load concentration was 4 pM (but it can be up to 8 pM) with a 10% PhiX spike to add diversity. Sequencing reagents were prepared according to Illumina’s protocol for 16S sequencing with the Illumina MiSeq personal sequencer (61). (Updated versions of this protocol can be found at http://www.mothur.org/wiki/MiSeq_SOP.) Custom read 1, read 2, and index primers were added to the reagent cartridge, and FASTQ files were generated for paired-end reads.

### Microbiome analysis.

Analysis of the V4 region of the 16S rRNA gene was done using mothur (version 1.33.3) (61, 62). Briefly, the standard operating procedure (SOP) at http://www.mothur.org/wiki/MiSeq_SOP was followed to process the MiSeq data. The paired-end reads were assembled into contigs and then aligned to the SILVA 16S rRNA sequence database (63, 64) and were classified to the mothur-adapted RDP training set v9 (65) using the Wang method and an 80% bootstrap minimum to the family taxonomic level. All samples with <500 sequences were removed. Chimeric sequences were removed using UCHIME (66). Sequences were clustered into operational taxonomic units (OTU) using a 3% species-level definition. The OTU data were then filtered to include only those OTU that made up 1% or more of the total sequences. The percentage of relative abundance of bacterial phyla and family members in each sample was calculated. A cutoff of 0.03 (97%) was used to define operational taxonomic units (OTU) and used to calculate the inverse Simpson index as a measure for diversity. Standard packages in R were used to create average heat maps per mouse treatment group and changes in the inverse Simpson index per treatment group (see Fig. S5 and S6 in the supplemental material).

### Correlation analysis.

We computed the Spearman’s rank correlation coefficients (*r*) for all pairs among the 26 bile acids and 121 OTU (Fig. 6) using “method = ‘spearman,’ use = ‘pairwise.complete.obs’” in R package as seen in reference 12.

### Statistical analysis.

Statistical tests were performed using Prism version 6.00d for Mac Os X (GraphPad Software, La Jolla, CA, USA). Significance between groups of *ex vivo* spore germination and outgrowth studies was done by Mann-Whitney nonparametric *t* test (Fig. 2). Significance between controls and all other groups in *in vitro* germination and growth studies were calculated by Student’s parametric *t* test (Fig. 4 and 5). The Kruskal-Wallis one-way analysis of variance test followed by Dunn’s multiple comparison test was used to calculate the significance of bacterial diversity of non-antibiotic-treated controls compared to all other groups in Fig. S6 in the supplemental material. Statistical significance was set at a *P* value of <0.05 for all analyses.

10.1128/mSphere.00045-15.7 Figure S6  Bacterial diversity of the murine ileal and cecal microbiome. Bacterial diversity of the (A) ileal and (B) cecal microbiota of all treatment groups measured by the inverse Simpson index. All treatment groups were compared to non-antibiotic-treated controls by Kruskal-Wallis one-way analysis of variance test followed by Dunn’s multiple comparison test. Each bar represents the mean ± SEM (*, *P* < 0.05; **, *P* < 0.01; ***, *P* < 0.001). Download Figure S6, EPS file, 1.5 MB. Copyright © 2016 Theriot et al. 2016 Theriot et al. This content is distributed under the terms of the Creative Commons Attribution 4.0 International license.
