# Supplementary figures and images for: Antibiotic-Induced Alterations of the Gut Microbiota Alter Secondary Bile Acid Production and Allow for Clostridium difficile Spore Germination and Outgrowth in the Large Intestine
Source: mSphere. 2016 Jan 6;1(1):e00045-15. doi: 10.1128/mSphere.00045-15 (PMC4863611; doi:10.1128/mSphere.00045-15)

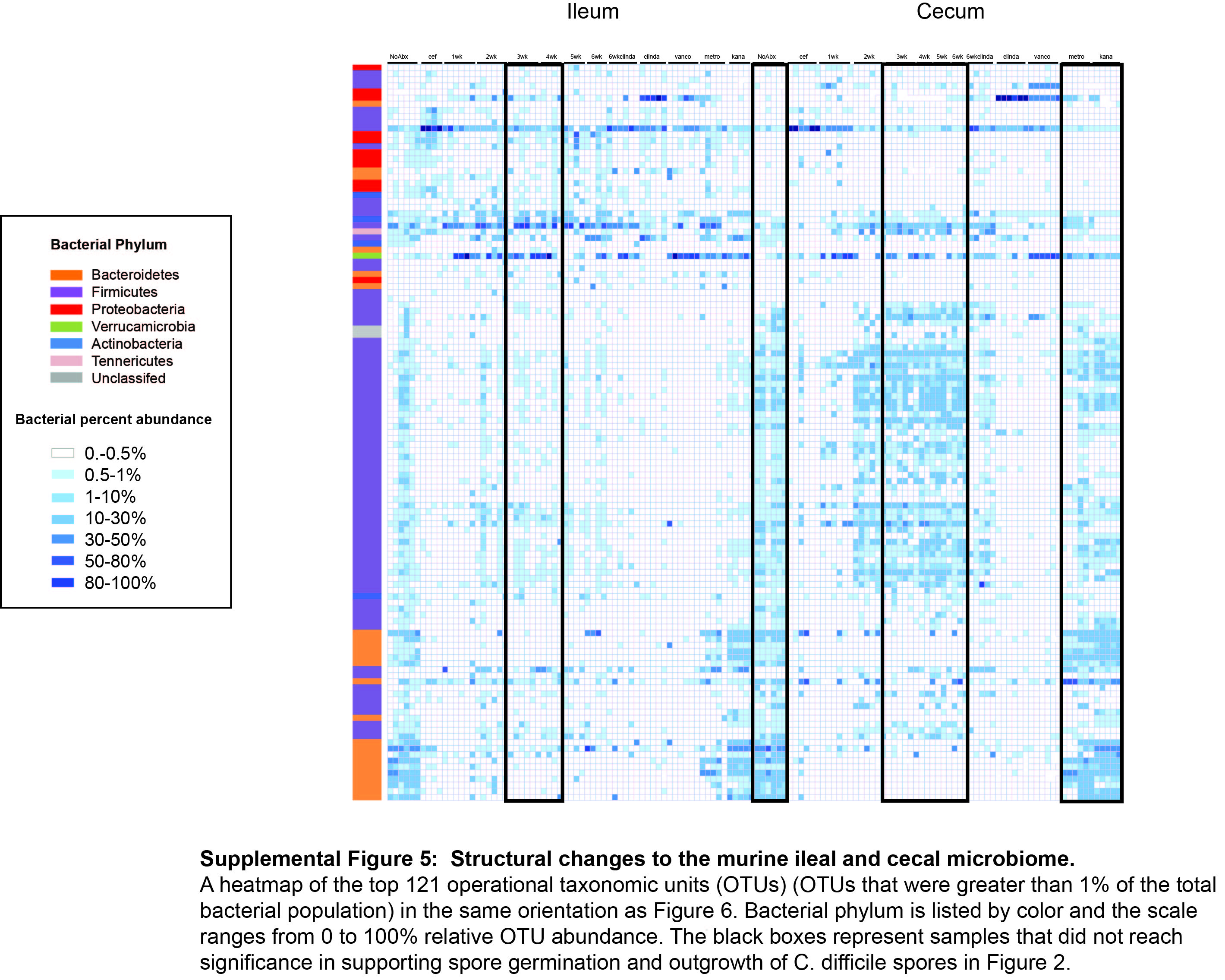

Supplement: Figure S5 [file sph001160046sf5.jpg]
